# Effect of a Tailored Activity Pacing Intervention on Fatigue and Physical Activity Behaviours in Adults with Multiple Sclerosis

**DOI:** 10.3390/ijerph18010017

**Published:** 2020-12-22

**Authors:** Ulric S. Abonie, Florentina J. Hettinga

**Affiliations:** 1 Department of Physiotherapy and Rehabilitation Sciences, University of Health and Allied Sciences, Ho, Volta Region PMB 31, Ghana; uabonie@uhas.edu.gh; 2 School of Sport, Rehabilitation and Exercise Science, University of Essex, Wivenhoe Park, Colchester CO4 3SQ, UK; 3 Department of Sport, Exercise and Rehabilitation, Northumbria University, Newcastle upon Tyne NE1 8ST, UK

**Keywords:** multiple sclerosis (MS), activity pacing, accelerometer, energy distribution

## Abstract

Tailored activity pacing could help manage fatigue and improve physical activity. However, little is known about how to tailor activity pacing for people with multiple sclerosis. This study aims to evaluate the effect of a tailored activity pacing intervention on fatigue and physical activity behaviours in adults with multiple sclerosis. Twenty-one adults with multiple sclerosis, stratified by age and gender, are randomly allocated to either a tailored pacing or control group. Participants wear an accelerometer for seven days that measures physical activity behaviours, and self-report fatigue at the baseline and four-week follow-up. Physical activity behaviours are assessed by examining activity level (seven-day average activity counts per minute) and activity variability (seven-day average highest activity counts each day divided by activity counts on that day). The intervention improves activity levels (Mean difference = 40.91; 95% Confidence Interval [CI] (3.84–77.96); *p* = 0.03) and lessens activity variability (Mean difference = −0.63; 95% CI (−1.25–0.02); *p* = 0.04). No significant effect is found for fatigue (Mean difference = −0.36; 95% CI (−1.02–0.30); *p* = 0.27). This investigation shows that tailoring activity pacing based on physical activity behaviours and fatigue is effective in improving physical activity levels, without exacerbating fatigue symptoms.

## 1. Introduction

Multiple sclerosis (MS) and its symptoms have been associated with a dramatic reduction in physical activity participation [1,2,3]. However, physical activity and participation are considered important health promoting behaviours to improve MS symptoms such as fatigue, quality of life, and maintenance of physical function [4,5,6]. This highlights the need to develop effective interventions for enabling physically active lifestyles in persons with MS. Activity pacing might present an efficacious and safe alternative to more traditional exercise programmes that could be inappropriate for individuals with MS, and could stimulate an active lifestyle in people with MS over time; such that their experience and expectations of fatigue in relation to engagement in physical activity are well-managed, and physical functioning is maintained [7,8].

Activity pacing is a strategy to divide one’s daily activities into smaller, more manageable portions, in a way that should not exacerbate their symptoms, which then allows gradual progressive increases in activity [9]. Instruction in activity pacing could help alter inefficient activity patterns such as being overactive (overdoing activity when feeling better in terms of reduced symptoms, consequently leading to exacerbation of symptoms followed by very prolonged inactive periods) or being under-active [7,8]. Our previous exploratory studies suggest that without interventions there appears to be no clear strategy amongst persons with MS to manage fatigue and improve physical activity, both in the short-term and long-term [7,10,11,12]. These findings suggest that guidance regarding the optimal use of pacing may be beneficial for persons with MS to improve their fatigue symptoms and physical activity behaviour.

Evidence of formal instruction in activity pacing is limited [7,8,9], however, a few studies have been done in populations other than those with MS [13,14,15], and most interventions target problematic symptoms that arise from overactivity [15,16,17]. This, however, represents a pitfall in the literature as underactivity also is linked to functional impairment [18]. Previous interventional studies that reported poor outcomes may have sampled from persons with prior underactive behaviour for whom instruction on activity pacing related to avoiding overactivity is likely to be non-beneficial, while positive outcomes may have been obtained from an overactive sample. Thus, interventions modelled on the assumption that overactivity needs to be prevented are less likely to be effective in underactive persons.

An individually-tailored activity pacing approach, based not only on a person’s symptom profile but, also, on their physical activity behaviour and attitudes toward physical activity, is needed to improve the success of activity pacing intervention. To our knowledge, no work has explored the effect of a tailored activity pacing approach based on attitudes and behaviour toward physical activity in promoting an active lifestyle and managing fatigue in people with MS. The aim of this study is to examine the effectiveness of a tailored activity pacing intervention based on individual physical activity patterns and fatigue experiences in lowering fatigue and improving physical activity levels and variability in adults with MS. We hypothesise the approach would decrease fatigue, increase activity levels, and decrease activity variability in people with MS.

## 2. Materials and Methods

### 2.1. Participants

Community-dwelling adult participants (age 59 ± 2 years) were recruited from the Multiple Sclerosis (MS)-United Kingdom (UK) centre and the MS Society focus group through public advertisements (online and e-posters) in Colchester, Essex. Interested participants were contacted by the researchers who explained the study rationale, potential benefits, and procedures, answered all questions, screened, and, in the event of meeting inclusion criteria and voluntarily agreeing to participate obtained a written informed consent. The study protocols were approved by the Ethics Board at the University of Essex (reference: 17/BS/499/AU). Participants were asked to inform researchers of change or initiation of medical or conservative treatment during the study period.

Participants were included if they were aged 18 years or older, had a definite diagnosis of MS, been relapse-free during the previous 30 days, ambulatory (with or without an assistive device), could reliably wear the accelerometer, and were English-speaking. People were excluded if non-ambulatory; experienced a relapse in the previous month; changed medications within the previous 2 weeks that could interfere with fatigue ratings or accelerometer data; and currently or recently (in the previous 12 months) received a physical activity programme with or without activity management instruction.

### 2.2. Procedure

**Figure 1:**
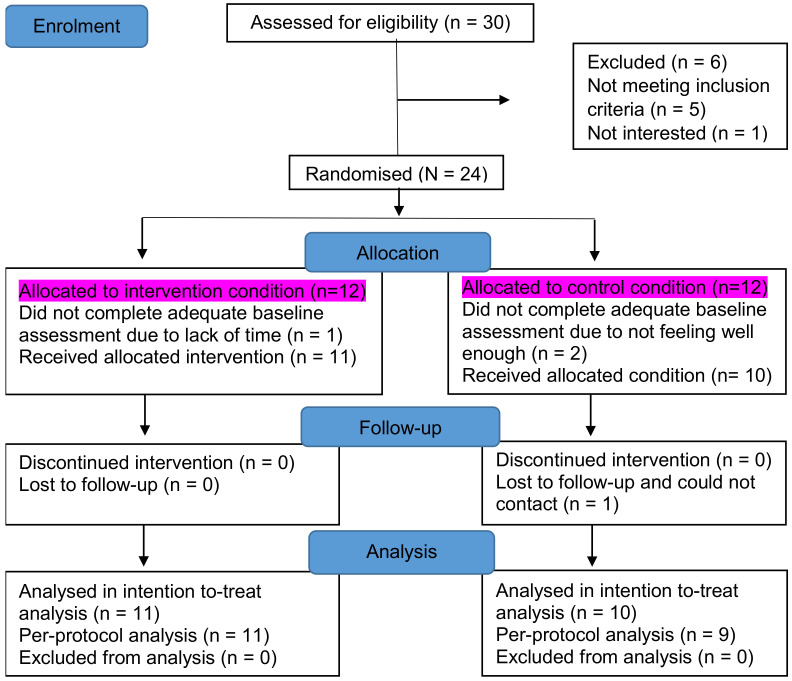
Flow diagram of participants through the intervention.

Recruitment for this study began in July 2017 and ended in December 2017. The main data collection periods were at the baseline and the 4-week follow-up. Occurring at the baseline, demographic, and health status information were collected. Background demographics included age, gender, Multiple Sclerosis (MS) diagnosis, duration of illness (years since diagnosis), body mass index, physical disability—assessed using the Patient Determined Disease Steps (PDDS) [19]—and health-related quality of life assessed with the RAND 12–Item Short-Form Health Survey Questionnaire [20]. Participants were then instructed to wear an accelerometer on their waist at all times except on occasions when it could become wet (e.g., showering or swimming), not to alter their activity behaviour, and keep an accompanying logbook to record daily fatigue, activity pacing behaviours and activities, in addition to wake-up and bedtimes, during a 7-day home monitoring period. After the home monitoring period, participants returned the accelerometer and logbook, were stratified by age and gender, and randomised into an intervention or control group. Participants blindly picked a folded paper marked with “intervention” or “control” out of a box. The intervention began within the week after the baseline assessment. Occurring at the 4 week follow-up, all participants wore the accelerometer for a 7-day home monitoring period and completed questionnaires. The flow chart of participants through the study and reasons for exclusions and withdrawals are displayed in Figure 1.

#### 2.2.1. Tailored Activity Pacing Intervention

Activity pacing was tailored based on data from the accelerometer and logbook which were used to generate personalised reports that summarised and visually depicted each person’s symptom-activity relationship based on their physical activity, fatigue, and physical activity patterns. This gave a representation of the person’s attitudes and behaviour toward physical activity such as avoidance behaviour and overactive behaviour.

Those whose report depicted activity avoidance in response to fatigue, or who were limiting their activities in the fear of a relapse evident in physical activity behavior demonstrating generally very low activity levels and moderately-severe fatigue ratings, were provided with information on perceptions and expectations with respect to activity-related symptoms and with strategies to develop graded consistent physical activity to increase their physical activity level and fitness.

Similarly, those whose report exemplified overdoing activities when feeling better, resulting in worsened fatigue, and then needing to rest for prolonged periods to recover—evident as low fatigue preceding high activity level clusters followed by severe fatigue and prolonged inactivity periods—were provided with information on developing a consistent pattern of paced activity and rest followed by a gradual increase in physical activity. The intervention session was approximately 30 min long—depending on the participant.

#### 2.2.2. Outcome Measures

##### Engagement in Pacing and Perceived Risk of Overactivity

Engagement in pacing and perceived risk of overactivity were assessed with the subscales of the Activity Pacing and Risk of Overactivity Questionnaire, as used in the study by Abonie et al., [10]. The questionnaire evaluated how and based on what aspects participants modified their Physical Activity (PA) behaviour over the day, which represented the participants’ attitudes and behaviours toward physical activity. Participants were asked to score each of the 7 items of the questionnaire on a scale of 1–5 (1, never; 2, rarely; 3, sometimes; 4, often; 5, very often). Two subscale scores: engagement in pacing score (1–5) and perceived risk of overactivity score (1–5) were calculated.

#### 2.2.3. Fatigue

Fatigue was measured using the Fatigue Severity Scale (FSS) [21]. The nine items were averaged to calculate a mean fatigue score that ranged from 1 (no fatigue) to 7 (very severe fatigue). The FSS has been proven to be a reliable and valid measurement tool to determine the impact of fatigue and to detect change over time [21,22]. A mean FSS score ≥ 4 was adopted as the cut-off for clinically significant fatigue, and a reduction of 0.5 points was considered to be clinically significant [23].

#### 2.2.4. Physical Activity

Physical activity was measured by a waist-worn accelerometer (ActiGraph GT3X+, LLC, Fort Walton Beach, FL, USA) [24]. Changes in acceleration were recorded into the accelerometer as activity counts, saved every 10 s, and then averaged each minute. Activity counts were computed based on pre-defined algorithm cut points (Freedson Adults VM3 [25]). Total physical activity was calculated by averaging the cumulative activity counts per minute over 7 days.

Besides the activity counts per minute, accelerometer data were used to calculate activity variability. Activity variability for each day was calculated as the amount of physical activity during the peak activity hour for each day (identified as the hour with the highest number of activity counts), divided by the mean amount of physical activity on that day, and averaged over 7 days. A high score indicated a high activity variability and a stronger concentration of physical activity, while a low score activity suggested a low variability and that physical activity was evenly spread throughout the day [14].

## 3. Statistics

All statistical analyses were performed using version 25.0 of the IBM Statistical Package for the Social Sciences software [26]. All values were reported using descriptive statistics of mean (M) ± standard deviation (SD) to summarise the characteristics of participants. An independent t test was used to determine baseline differences between groups. The effect of the intervention on fatigue severity, activity level, and activity variability between the two groups was evaluated with a 2 factor repeated measures analysis of variance. The difference between the baseline (pre-test) and 4-week follow-up (post-test) was used as a “within-subject factor” and the group as a “between-subjects factor”. Regarding the intention-to-treat analyses, all eligible participants were included and missing data were carried forward from earlier results (notionally designating conservative outcomes of non-improvement over time). The significance level was set at *p* < 0.05 for all tests. Data were checked for normality using the Shapiro–Wilk test and visually inspecting Q–Q plots. The data were generally normally distributed.

Since the small sample size likely affected the power to detect small-to-moderate effects, the effect size d also was presented for these analyses [27]. Effect sizes were calculated as the square root of the partial eta-squared, divided by one minus the partial eta-squared, multiplied by two (square root (partial eta-squared/(1—partial eta-squared)) × 2) [27] and interpreted according to Cohen’s d [27]. Effect sizes of 0.20 were considered small, 0.50 moderate, and 0.80 large.

## 4. Results

### 4.1. Participant Characteristics

Among the 30 individuals who were identified as eligible to participate, 24 were randomly assigned to either the intervention or the control group, and 21 were recruited into the study with adequate baseline measures completed (intervention group: n = 11; control group: n = 10). Demographics and baseline measures of the participants are presented in Table 1.

The sample (*n* = 21) was 71% male, and the mean age was 59.3 ± 1.9 years. Seventy-six percent of the sample had clinically significant fatigue. There were no significant differences between the groups at the baseline; however, compared with the control group, participants in the intervention group were slightly younger (57.9 ± 8.0 versus 60.9 ± 9.5 years, *p* = 0.44). Fatigue, physical activity level, and physical activity variability were similar in the intervention and control groups: 4.7 ± 2.0 versus 4.8 ± 1.2, *p* = 0.88; 296.5 (149.2) versus 195.2 (131.7), *p* = 0.06; and 4.0 ± 0.9 versus 3.9 ± 0.5, *p* = 0.87, respectively. Their health-related quality of life was similar (intervention: 43.0 ± 8.6 versus control: 42.3 ± 8.0, *p* = 0.84). Two participants discontinued due to illness, work, or family commitments. One participant completed the baseline measures only, allowing an intention-to-treat analysis to be conducted.

### 4.2. Influence of Tailored Activity Pacing Intervention

A clinically-significant improvement in fatigue was observed in 2 of 11 participants in the tailored activity pacing intervention group (mean differences of 1.67 and 1.22) compared to 1 of 10 in the control group (mean difference = 1.11) at the 4–week follow-up. Two participants in the control group reported an increase in their Fatigue Severity Scale (FSS) score by 0.5 points at the 4–week follow-up, compared to only one participant in the tailored activity pacing intervention group. Comparisons of fatigue severity, activity level (counts per minute), and activity variability before and after the intervention are presented in Table 2.

### 4.3. Physical Activity

Significant group × time interactions were found, showing an increase in activity level (Mean difference = 40.91; 95% Confidence Interval [CI] (3.84–77.96); *p* = 0.03) and a lessened activity variability (a more even spread of activities throughout the day) (Mean difference = −0.63; 95% CI (−1.25–0.02); *p* = 0.04). The effect sizes on activity level and activity variability were large (1.06 and 0.99, respectively). No significant main effects of time were found in activity level (counts per minute) and activity variability (*p* > 0.05).

### 4.4. Fatigue

No significant interaction or main effect of time were detected in fatigue severity (Mean difference = −0.36; 95% CI (−1.02–0.30); *p* = 0.27).

**Figure 2:**
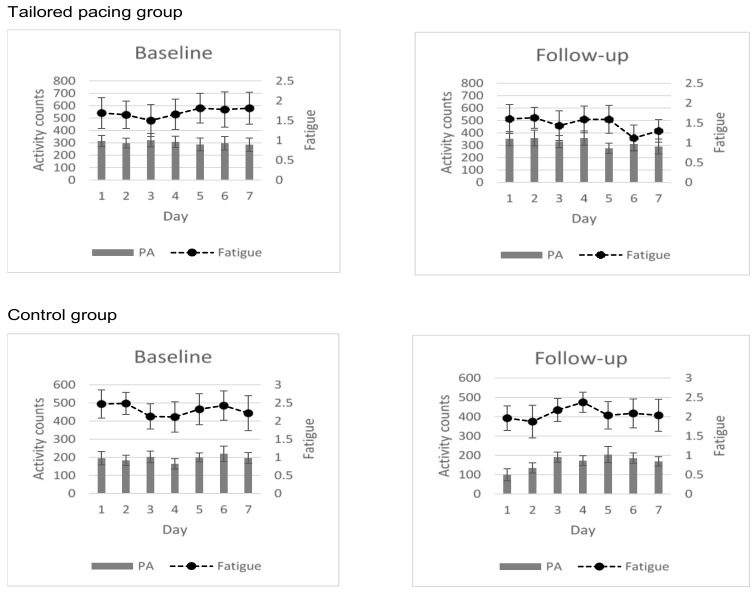
Changes in fatigue (dashed line) and activity levels; counts per minute (grey bar) between baseline and follow-up over a 7-day period.

Plotting of the mean fatigue score and activity level of all participants during the baseline and follow-up home monitoring periods (Figure 2), revealed positive changes in fatigue severity and activity levels in favour of the tailored activity pacing group compared to the control.

## 5. Discussion

This study examined the effectiveness of a tailored activity pacing intervention based on personalised reports that summarised and depicted a person’s attitudes and behaviour toward physical activity and fatigue experiences, in improving fatigue and physical activity in adults with Multiple Sclerosis (MS). The approach was effective in improving activity levels and activity variability, and findings support our hypothesis that guidance on activity pacing would increase activity levels and decrease activity variability in people with MS. The study findings were unlikely to be biased by the fluctuating nature of the health status of people with MS. Indeed, the outcome of the comparison of the data generated during the first and final home monitoring was confirmed by the analysis (Table 2) and plotting (Figure 2) of fatigue and physical activity of all participants at the baseline and 4-week follow-up.

Regarding physical activity, a significant interaction and a large effect was detected in activity levels measured in counts per minute with a waist worn accelerometer. Other studies investigating the effect of activity pacing did not find these beneficial effects on activity levels [13,14]. It is worth mentioning that most of these studies aimed the activity pacing intervention and guidance only at preventing overactivity and fatigue symptom exacerbation. Therefore, the large effect on activity levels obtained in this study points to the important role of tailoring activity pacing interventions and advice to a person’s characteristics to achieve beneficial health outcomes for all. It also highlights the need to not only tailor activity pacing interventions based on fatigue experiences, but also based on attitudes and behaviour toward physical activity measured at the baseline.

Additionally, a significant interaction and a large effect was found showing a decrease in physical activity variability. This indicates that the intervention resulted in a more even spread of activities throughout the day. This study is the first to show that tailored activity pacing was effective in improving activity levels and variability in persons with MS. Previous works regarding adults with osteoarthritis found a beneficial effect on activity variability but not activity levels [13]. Our tailored activity pacing intervention specifically targeted a behavioral change by attempting to spread the amount of physical activity throughout the day and stabilise the fluctuating nature of the physical activity pattern throughout the week. Given that the tailored intervention was designed to target inefficient activity patterns from the home monitoring period, it is not surprising and is worth mentioning that physical activity levels and physical activity variability were most greatly impacted in the tailored activity pacing intervention, as shown in Figure 2, and the large significant beneficial effect sizes for activity levels (1.06) and activity variability (0.99).

The large beneficial effect sizes coupled with the statistically significant finding for physical activity levels and physical activity variability throughout the week provided valuable insights. This indicated the effectiveness of a tailored activity pacing intervention to stimulate an active lifestyle in persons with MS and suggests that tailored activity pacing may need to be incorporated in activity stimulation programmes for people with MS to help them remain or become active. These findings also suggest that previous activity pacing interventions that had negative outcome effects may have focused largely on preventing overactivity and symptom exacerbation in a sample that may have been exhibiting avoidance behaviour toward physical activity. Although most of these interventions target problematic symptoms that arise from overactivity, none evaluated perceived risk of overactivity in daily life and the subsequent influence of the intervention on perceived risk of overactivity. Exploring this seems necessary to identify and characterise the target population and help adapt interventions for successful outcomes.

The current study tailored activity pacing to the person’s attitudes toward activity pacing and physical activity, as an improvement to the other research, in terms of a person’s fatigue experience and physical activity patterns assessed at the baseline. The increase in physical activity levels and the decrease in activity variability without exacerbation of fatigue symptoms found in this study supports the need to disassociate symptoms from activity when tailoring pacing, so behaviours are not a reactionary response to increased symptoms but rather an anticipatory strategy [28,29]. Conversely, the increase in physical activity levels and the decrease in activity variability without exacerbation of fatigue found in this study points to the fact that by perceiving and/or noticing an increase in physical activity without a worsening of fatigue symptoms, persons with MS are likely to feel more in control and focus less on their fatigue, which can lead to positive effects such as better fatigue management and activity participation as well as engagement in a physically active lifestyle in persons with MS.

Regarding fatigue, despite the results not reaching statistical significance, there appears to be a trend toward a decrease in fatigue severity in the tailored pacing group, similar to that found in adults with chronic fatigue syndrome [14,30] and osteoarthritis [13]. Conversely, the improvement in physical activity level and variability without worsening fatigue severity provides further insight into the beneficial effects of tailored activity pacing.

The small sample size, atypical high proportion of males and older people with MS in this pilot study limits our ability to generalise the findings. Also, the study inclusion criterion of only ambulatory persons may have excluded people who were severely affected by MS (i.e., mostly wheelchair dependent or bedridden) from participating. Including these people in future research is warranted. Furthermore, additional treatment sessions would be required to provide a comprehensive programme, and a longer follow up will provide more insight into the benefits of tailored activity pacing for those with MS. Replicating the study in a larger sample is warranted to examine subject variables that may moderate the effects of the tailored activity pacing intervention and allow firm conclusions.

## 6. Conclusions

This study showed that tailoring activity pacing to individuals’ attitudes and behaviours toward physical activity and their fatigue experiences, targeting an even spread of daily activities, has a significantly large beneficial effect upon physical activity levels and variability in activity levels in adults with Multiple Sclerosis (MS). These preliminary findings are very promising, and this study shows the short-term benefits of a tailored activity pacing intervention on activity levels and variability, without a worsening of fatigue for people with MS. Findings call for a larger study, including a longer follow-up assessment period to evaluate the medium term effects of the tailored activity pacing intervention for people with MS. This low-resource intervention looks promising for the management of fatigue and stimulation of an active lifestyle. The study findings provide the basis for incorporating tailored activity pacing in physical activity promotion programmes to help people with MS remain or become active.

## Figures and Tables

**Table 1:** Descriptive Characteristics of Participant.

Variable	Tailored Information Group	Control Group	*p*
Number of participants	11	10	
Age, year (M ± SD)	57.9 ± 8.0	60.9 ± 9.5	0.444
Body mass index, kg/m^2^ (median, IQR)	25.2 (3.9)	25.1 (7.6)	0.310
Gender, No. of males (%)	8 (73)	7 (70)	0.897
MS type, No. of RRMS (%)	6 (60)	4 (40)	0.610
No. of PPMS (%)	1 (50)	1 (50)	
No. of SPMS (%)	4 (55)	5(50)	
Disease duration, year (median, IQR)	12.0 (24.0)	9.5 (19.5)	0.551
PDDS disability scale (median, IQR)	2.0 (2.0)	3.5 (2.0)	0.727
Fatigue severity (M ± SD)	4.7 ± 2.0	4.8 ± 1.2	0.876
Activity level, cpm (median, IQR)	296.5 (149.2)	195.2 (131.7)	0.063
Activity variability (M ± SD)	4.0 ± 0.9	3.9 ± 0.5	0.869
Health-related quality of life (M ± SD)	43.0 ± 8.6	42.3 ± 8.0	0.840
Engagement in Pacing (M ± SD)	3.2 ± 0.8	3.2 ± 0.7	0.965
Perceived risk of overactivity (M ±SD)	3.5 ± 1.3	3.2 ± 0.7	0.454

cpm = counts per minutes; IQR = interquartile range; M = mean; min/wk = minutes per week; MS = Multiple Sclerosis; PA = Physical activity; PDDS = Patient Determined Disease Steps; PPMS = Primary Progressive MS; RRMS = Relapsing Remitting MS; SD = standard deviation; SPMS = Secondary Progressive MS.

**Table 2:** Changes in outcomes between baseline and follow up.

	Intervention Group (*n* = 11)		Control Group (*n* = 10)		Pre-Post Test	Interaction (Group*Time)		
Outcome	Pre-Test	Post-Test	Pre-Test	Post-Test	*p*	*p*	F	d
Fatigue severity	4.7 ± 2.0	4.6 ± 1.9	4.8 ± 1.2	5.1±1.1	0.91	0.27	1.27	0.52
Activity level (cpm)	308.5 ± 151.6	331.3 ± 156.9	202.4 ± 80.0	184.3 ± 71.4	0.79	0.03	5.34	1.06
Activity variability	4.0 ± 0.9	3.9 ± 1.0	3.9 ± 0.5	4.5 ± 0.9	0.16	0.04	4.66	0.99

Values are presented as mean ± standard deviation; Con = control; cpm = counts per minute; d = Cohen’s d effect size; Int = intervention; n = number of participants; PA = physical activity.

## Data Availability

The data presented in this study are available on request from the corresponding author. The data are not publicly available due to privacy issues.
